# Temporal Dynamics of Retinal Inflammation Following Blast Exposure in a Ferret Model

**DOI:** 10.1089/neur.2024.0127

**Published:** 2025-04-09

**Authors:** Rex Jeya Rajkumar Samdavid Thanapaul, Chetan Pundkar, Gaurav Phuyal, Manoj Y. Govindarajulu, Ashwathi Menon, Joseph B. Long, Peethambaran Arun

**Affiliations:** ^1^ Blast-Induced Neurotrauma Branch, Center for Military Psychiatry and Neuroscience, Walter Reed Army Institute of Research, Silver Spring, Maryland, USA.; ^2^ National Research Council (NRC) Research Associateship Programs, National Academies of Sciences, Engineering, and Medicine, Washington, District of Columbia, USA.; ^3^ Oak Ridge Institute for Science and Education (ORISE) Fellowship, Oak Ridge, Tennessee, USA.; ^4^ The Army Educational Outreach Program (AEOP), Walter Reed Army Institute of Research, Silver Spring, Maryland, USA.; ^5^ College of Natural and Mathematical Sciences, Baltimore County, University of Maryland, Baltimore, Maryland, USA.

**Keywords:** blast-induced traumatic ocular injury, cyclooxygenase, ferrets, inflammation, retinal damage, Toll-like receptors

## Abstract

Blast-induced traumatic ocular injury (bTOI) is a major cause of vision loss in military personnel involved in recent combat operations. However, its underlying mechanisms remain poorly understood, hindering the development of effective treatments. This study investigated the temporal expression patterns of key inflammatory markers in the retina after blast exposure using a ferret model. Ferrets (*n* = 40) were subjected to two tightly coupled blasts (20 psi) using an advanced blast simulator. Retinal tissues were collected at 4 h, 24 h, or 28 days post-blast. Differential mRNA expression of Toll-like receptors (*TLR*s: *1–9*), cytokines (*IL*: *1β, 6,* and *10*), and cyclooxygenase enzymes (*COX*: 1 and 2) was assessed using quantitative real-time polymerase chain reaction after blast exposure and compared with sham controls. Our results revealed a rapid and sustained upregulation of multiple *TLR*s (1, 2, 4, 5, 7, and 8) in the retina following blast exposure, indicating a robust inflammatory response. This was accompanied by a significant increase in pro- and anti-inflammatory cytokines (*IL-1β*, *IL-6 IL-10,* and *COX2*) at 4 h post-blast, suggesting their involvement in the acute pathogenesis of bTOI. Our findings emphasize the critical role of early innate immune responses and the potential for chronic inflammation in bTOI, highlighting the importance of timely therapeutic interventions. Targeting these inflammatory pathways may offer therapeutic avenues for mitigating retinal damage and improving ocular function.

## Introduction

Blast-induced traumatic ocular injuries (bTOI) pose a significant threat to both military and civilian populations, often leading to irreversible visual impairment and blindness.^1^ Recent reports from conflict zones like Gaza highlight the ongoing global concern about ocular injuries in military operations, with at least 15% of Israeli Defense Forces soldiers having suffered significant ophthalmological injuries resulting in vision loss.^2^ Similarly, ocular trauma, primarily due to blast injuries (64–84%) from improvised explosive devices, has been a leading cause of injury among U.S. service members during the Iraqi Freedom and Afghanistan Enduring Freedom conflicts, accounting for 51–69% of all ocular injuries.^3–6^ The prevalence of open and closed-globe injuries emphasizes the severity of bTOI and its devastating impact on vision.^7^

The incidence of penetrating versus nonpenetrating ocular injuries in humans after blast injuries varies depending on the nature of the explosion, proximity to the blast, and environmental factors. Penetrating injuries account for approximately 30–50% of blast-induced ocular injuries in conflict zones, typically resulting from high-velocity projectiles such as shrapnel, debris, or glass fragments caused by secondary and tertiary blast effects.^8,9^ These injuries tend to be more severe, with a higher risk of permanent vision loss or blindness, often requiring surgical intervention and carrying a significant risk of complications such as endophthalmitis.^4,10^ In contrast, nonpenetrating injuries, including globe contusions, lens dislocations, retinal detachments, and vitreous hemorrhages, are slightly more common, comprising 50–70% of ocular injuries in blast scenarios.^8,11^ These injuries are usually caused by the primary blast wave’s force or blunt trauma due to the tertiary effects of the explosion.

Ocular dysfunction and structural damage are frequently intertwined with traumatic brain injury (TBI), another common consequence of blast exposure.^12,13^ Primary blast waves can directly damage ocular tissues and visual pathways, while secondary injuries caused by flying debris further exacerbate the problem.^14^ Importantly, repeated exposure to blasts, whether low-intensity in military training exercises or high-intensity on the battlefield, can lead to both acute and chronic ocular damage.

Nonpenetrating injuries, such as blast-TBI (bTBI), often result in retinal damage caused by shear forces and inflammation, accompanied by neuroinflammatory responses mediated by Toll-like receptors (TLRs) and cytokine signaling pathways.^15^ Unlike rodent models, ferrets possess gyrencephalic brains that closely resemble human brains, a key advantage when studying the visual system. This similarity is particularly significant because retinal signals are processed in distinct brain regions, and the ferret’s gyrencephalic brain better replicates the human brain’s ability to integrate, interpret, and respond to retinal inputs. These anatomical and functional parallels position ferrets as an invaluable translational model for investigating the effects of injuries like bTBI on retinal and cortical structures, as well as the resulting neuroinflammatory processes.

The high prevalence and economic burden of vision impairments among military personnel with TBI underscore the critical need to address bTOI.^16^ Research has demonstrated that the immune response plays a crucial role in exacerbating bTOI,^17^ and ocular dysfunction can persist for years after combat-related TBI.^13,18^ Despite the prevalence of bTOI and its lasting consequences, the molecular mechanisms underlying this injury, primarily caused by blast overpressure waves, remain poorly understood. This knowledge gap hinders the development of targeted therapies.

The immune response, involving activation of inflammatory *TLR*s, cyclooxygenase (*COX*) enzymes, and proinflammatory interleukins (ILs), is pivotal in exacerbating this damage after TBI.^19^ For instance, *TLR* activation triggers oxidative stress and neuroinflammation after TBI,^20^ and drugs targeting specific inflammatory markers have shown efficacy in animal models of non-blast-related brain injuries.^21^ *COX* enzymes, central to prostaglandin synthesis and inflammatory responses, warrant further investigation regarding their expression patterns and potential interaction with *TLR* signaling in the central nervous system, particularly the retina, post-blast. Similarly, *IL*s, a family of pro- and anti-inflammatory cytokines, play critical roles in immune responses and inflammation. However, the expression and regulation of specific *IL*s in the central nervous system, including the ocular signal processing system, following blast exposure remain incompletely understood. Understanding the interplay between *IL*s and other inflammatory pathways, such as *TLR* signaling, could uncover novel therapeutic targets for mitigating neuroinflammation and retinal damage post-blast.

To address this, we hypothesize that blast overpressure induces a time-dependent expression of inflammatory markers in the retina. In this study, we used a ferret model of bTOI to investigate the expression patterns of inflammatory markers in the retina following blast exposure.

## Methods

All animal procedures followed an Institutional Animal Care and Use Committee (IACUC)-approved protocol, adhering to the Animal Welfare Act and guidelines outlined in the Guide for the Care and Use of Laboratory Animals. Adult male ferrets (*Mustela putorius furo*), aged 13–15 weeks and weighing 1–1.2 kg, were acquired from Triple F Farms Inc, PA, USA. They were pair-housed in ventilated cages under controlled environmental conditions (20–22°C) with a 12-h light/dark cycle and provided *ad libitum* access to food and water throughout the study.

Ferrets were randomly assigned to either a sham group or a repeated blast group, with a minimum of four ferrets per group per time point (*n* = 40 total). Following anesthesia with 5% isoflurane for 8 min, the ferrets were secured in a longitudinal prone position using a custom-designed holder in the test section (2′ × 2′ inner dimensions) of an advanced blast simulator (ABS)^22^ to ensure stability while facing the oncoming shockwave. The ABS features a 0.5-ft long compression chamber separated from a 21-ft long transition/expansion test section by rupturable Valmex® membranes (Mehler Technologies, VA, USA). To induce moderate bTOI, animals in the blast group were exposed to two tightly coupled blast overpressure waves, spaced 2 min apart, yielding a peak positive static pressure of 20 psi and a positive phase duration of 4–5 ms, as previously described.^22,23^ Sham group animals were anesthetized and placed in the recovery cage without blast exposure. Animals were humanely euthanized using a 5% isoflurane overdose in compliance with Walter Reed Army Institute of Research (WRAIR)-IACUC guidelines at 4 h, 24 h, or 28 days post-blast. Retinal tissues were promptly collected and preserved in RNAprotect® (Qiagen Sciences, MD, USA).

Total retinal RNA was extracted using the RNeasy Kit (Qiagen Sciences, MD, USA). RNA quantity and purity were assessed using a Nanodrop D2000 (Thermo Scientific, MA, USA), with samples exhibiting an absorbance ratio (260/280 nm) of 1.8–2.0 were reverse transcribed into cDNA using the ZymoScript RT PreMix Kit (Zymo Research, CA, USA). Gene expression analysis was performed using quantitative real-time polymerase chain reaction (qRT-PCR) with RT^2^ SYBR Green qPCR Mastermix (Qiagen Sciences Inc, MD, USA) on an Applied QuantStudio 6 Flex qPCR system (Life Technologies, CA, USA), with each sample run in triplicate. Relative expression of target genes (*TLRs 1–9*, *COX1* & *2*, *IL-1β*, *IL-6*, and *IL-10*) was normalized to β-actin and calculated using the 2^−ΔΔCt^ method. Primer sequences of *TLR*s, as previously described,^23^ were used and for *IL-1β*: (F: 5′-FTTTCTAAAGCAGCCATGGCA-3′; R: 5′-CTTCTACTCCCT TTCCATCAG-3′), *IL-6*: (F: 5′-CAAATGTGAAGACAGCAAGGAGGCA-3′; R: 5′-TCT GAAACTCCTGAAGACCGGTAGTG), *IL-10*: (F: 5′-TCCTTGCTGGAGGACTTTAAGGGT-3′; R: 5′-TCCACCGCCTTGCTCTTATTCTCA-3′), *COX1*: (F: 5′-CATCCATCTACT CCCAGAGTCATGAG-3′; R: 5′-GAGGGCTGGGGATAAGGTTGGACCGC-3′), *COX2*: (F: 5′-GATTGACAGCCCACCAACTT-3′; R: 5′-CGGGATGAACTCTCTCCTCA-3′) were used.

Statistical analyses were performed using GraphPad Prism version 9.5.1.733 for Windows (GraphPad Software, CA, USA). An unpaired *t-test* was employed to assess the effects of blast exposure compared to sham animals, with statistical significance set at *p* < 0.05.

## Results

**FIG. 1.**
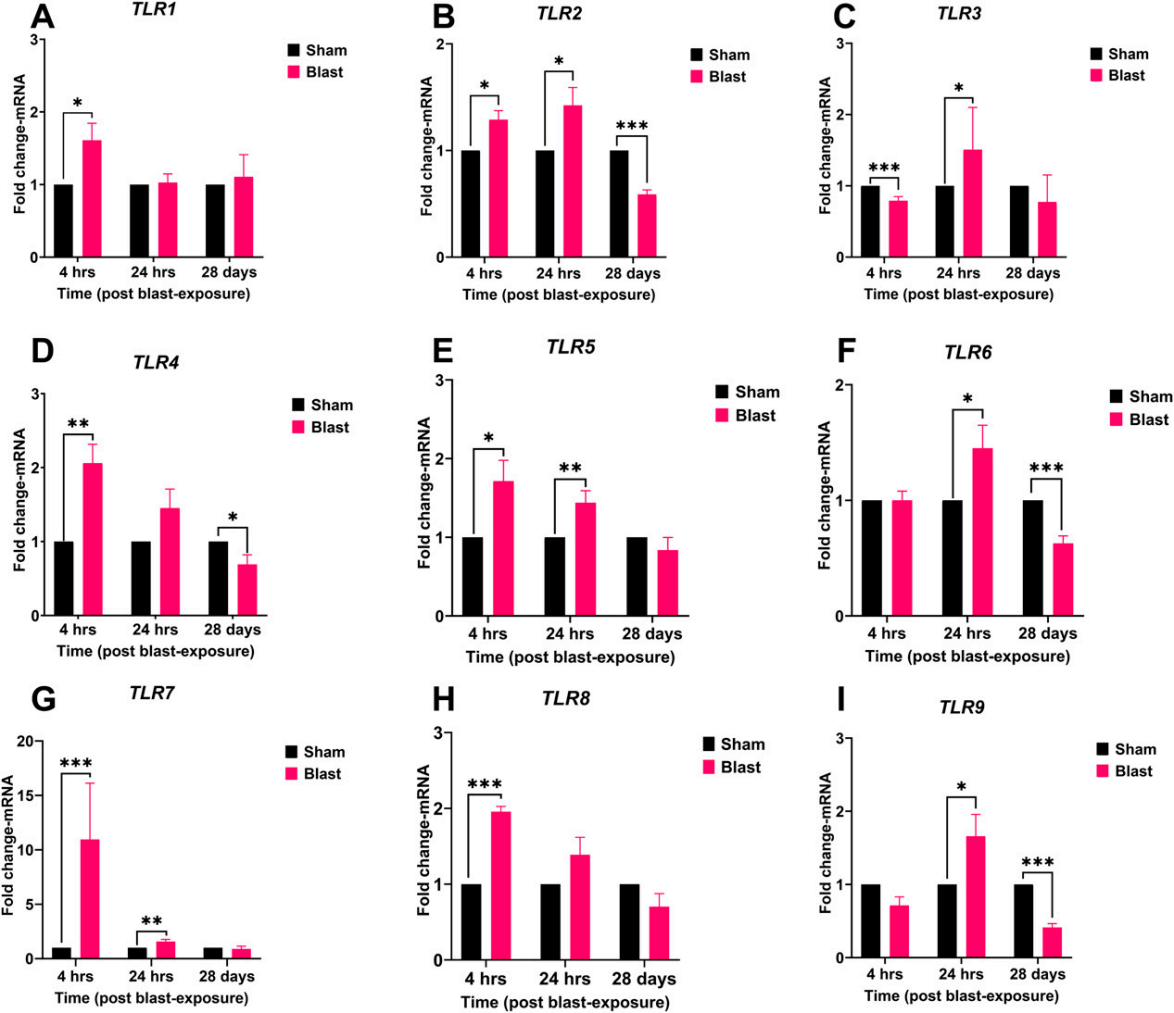
Blast exposure induces differential expression of Toll-like receptors (*TLR*s) in the ferret retina. Retinal tissue from sham (black bars) and blast-exposed (magenta bars) ferrets were collected at 4 h, 24 h, or 28 days post-exposure. mRNA expression of *TLR1–9* **(A–I)** was quantified by qRT-PCR and presented as fold change relative to the sham group at each time point (mean ± SEM). Sample size (*n*): 4 animals/group/time point (4 h) and 8 animals/group/time point (24 and 28 days). Statistical analysis: *t-test*. Asterisks indicate significant differences compared with the sham group (*p < 0.05, **p < 0.01; ***p < 0.001). qRT-PCR, quantitative real-time polymerase chain reaction; SEM, standard error of mean.

Blast exposure triggered a rapid and widespread upregulation of *TLR*s in the retina (Fig. 1A–I). At 4 h post-blast, statistically significant increases were observed in *TLR1* (1.61-fold, *p* = 0.039), *TLR2* (1.28-fold, *p* = 0.014), *TLR4* (2.06-fold, *p* = 0.004), *TLR5* (1.71-fold, *p* = 0.035), *TLR7* (10.95-fold, *p* < 0.001), and *TLR8* (1.95-fold, *p* < 0.001) mRNA expression compared with the sham group. This heightened *TLR* expression largely persisted at 24 h post-blast, with sustained upregulation of *TLR2* (1.42-fold, *p* = 0.018), *TLR3* (1.5-fold, *p* = 0.031), *TLR5* (1.43-fold, *p* = 0.009), *TLR6* (1.45-fold, *p* = 0.031), *TLR7* (1.57-fold, *p* = 0.007), and *TLR9* (1.65-fold, *p* = 0.045). By 28 days post-blast, a significant decrease in the expression of mRNAs for *TLR2* (*p* < 0.001), *TLR4* (*p* = 0.023), *TLR6* (*p* < 0.001), and *TLR9* (*p* < 0.001) was observed.

**FIG. 2.**
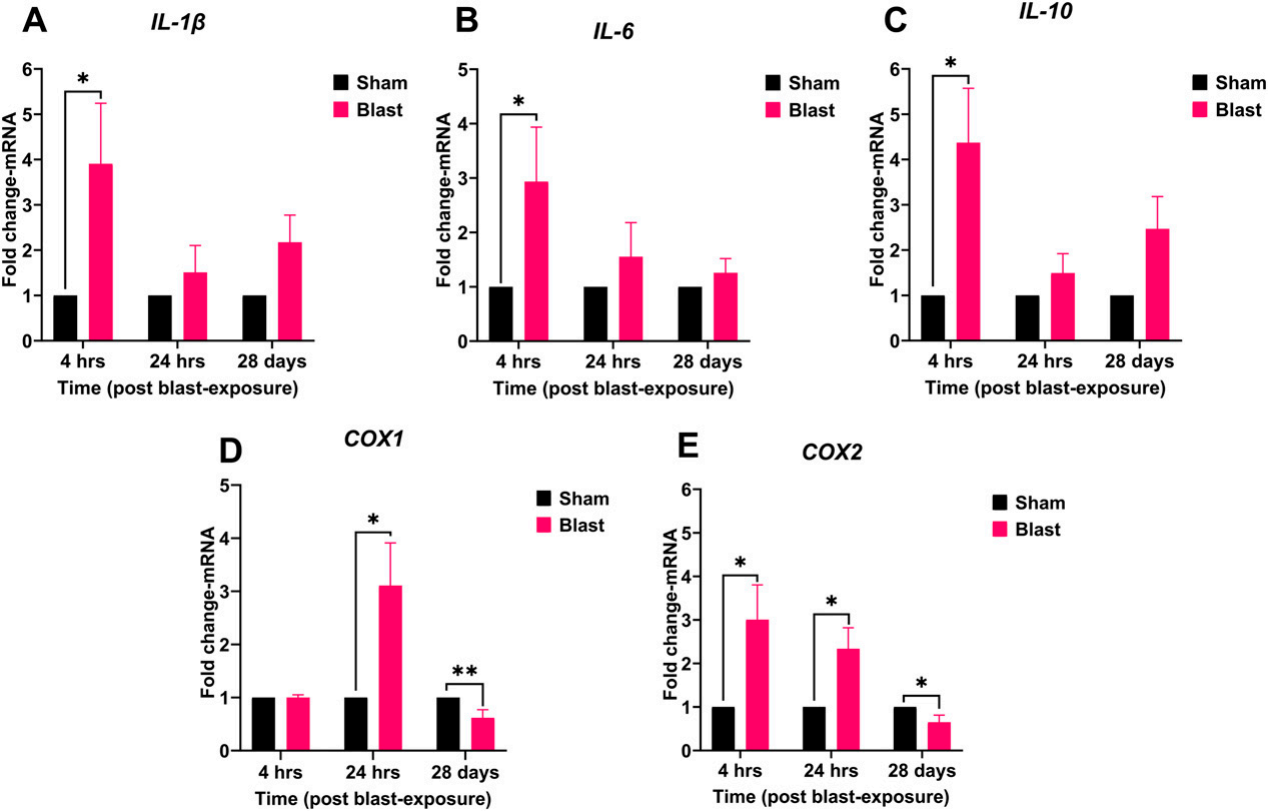
Blast exposure induces differential expression of cytokines and cyclooxygenase enzymes in the ferret retina. Retinal tissue from sham (black bars) and blast-exposed (magenta bars) ferrets were collected at 4 h, 24 h, or 28 days post-exposure. mRNA expression of *IL-1β*, *IL-6*, *IL-10*, *COX1*, and *COX2* **(A–E)** was quantified by qRT-PCR and presented as fold change relative to the sham group at each time point (mean ± SEM). Sample size (*n*): 4 animals/group/time point (4 h) and 8 animals/group/time point (24 and 28 days). Statistical analysis: *t-test*. Asterisks indicate significant differences compared with the sham group (^*^p < 0.05, ^**^p < 0.01). *COX*, cyclooxygenase; *IL*, interleukin; qRT-PCR, quantitative real-time polymerase chain reaction; SEM, standard error of mean.

Blast exposure also caused a robust inflammatory response in the retina (Fig. 2A–E). At 4 h, the pro-inflammatory cytokine *IL-1β* (3.90-fold, *p* = 0.048) was significantly upregulated, as was cyclooxygenase enzyme *COX2* (3-fold, *p* = 0.031). At 24 h, persistent inflammation was observed with statistically significant and sustained upregulation of *COX1* (3.10-fold, *p* < 0.02) and *COX2* (2.33-fold, *p* = 0.07). The long-term effects of blast exposure on retinal gene expression were not evident at 28 days for *IL-1β*, *IL-6,* and *IL-10*; however, there was a significant decrease in *COX1* and *COX2* levels.

## Discussion

Blast exposure can trigger a complex cascade of events in the retina, including inflammation and neuronal damage. Preliminary studies in animal models have shed light on the consequences of ocular trauma, revealing disruption of neuronal integrity, activation of glial cells, and damage to retinal neurons following blast exposure.^24–31^ These findings highlight the vulnerability of the visual system to explosive forces. Retinal injury triggers a complex molecular interplay, with multiple *TLR*s, cyclooxygenase enzymes (*COX1* and *COX2*), and cytokines like *IL-1β* and *IL-6* driving inflammation and damage, while *IL-10* counterbalances this response and promotes protection/healing. Understanding these mechanisms and their timing is crucial for developing targeted therapies to protect and restore vision after retinal injury.

Our study comprehensively analyzed the temporal expression patterns of the retinal inflammatory response following blast exposure using a ferret model. Due to its anatomical, physiological, and susceptibility similarities to the human nervous system,^32,33^ this model is a valuable system for studying the molecular mechanisms of bTOI and investigating potential therapeutic interventions. Although data on retinal injuries in ferret models of blast trauma are limited, our findings address this gap by providing the first time-resolved analysis of key inflammatory markers in the retina post-blast. These findings validate the ferret as a relevant model for bTOI studies and enhance our understanding of retinal responses to blast injuries, bridging insights from rodent models to a system more aligned with human clinical scenarios. To our knowledge, this is the first study to comprehensively assess time-dependent and retina-specific mRNA expression changes in key inflammatory markers within a clinically relevant bTOI model. Following blast exposure, we observed dynamic changes in retinal *TLR*s and genes related to inflammatory and oxidative stress responses, underscoring the ferret’s utility in elucidating the molecular foundations of bTOI.

The rapid and sustained upregulation of *TLR*s post-blast indicates a robust inflammatory response in the retina. Significant increases in *TLR1*, *TLR2*, *TLR4*, *TLR5*, *TLR7*, and *TLR8* at 4 h post-blast suggest acute innate immune response activation, playing critical early roles. The persistent upregulation of *TLR*s at 24 h, mainly *TLR2*, *TLR3*, *TLR5*, *TLR6*, *TLR7*, and *TLR9*, points to ongoing inflammation. The temporal responses of several *TLR*s suggest complex regulatory mechanisms that balance pro- and anti-inflammatory signals at acute and sub-acute time points. By 28 days, most *TLR* expressions returned to baseline, indicating acute inflammation resolution, while sustained downregulation of some *TLR*s suggests potential long-term suppression due to endogenous defensive mechanisms. This differential *TLR* regulation underscores the importance of temporal dynamics in the immune response. Early upregulation likely triggers downstream inflammation, while later resolution indicates attempted homeostasis restoration. The acute upregulation of different *TLR*s suggests that therapeutic agents that can inhibit multiple *TLR*s more effectively protect against bTOI than those that can inhibit only a specific *TLR*.

The significant upregulation of pro-inflammatory cytokines *IL-1β* and *IL-6*, along with *IL-10* and *COX2*, at 4 h post-blast indicates a robust early inflammatory and oxidative stress response. The subsequent *COX1* increase at 24 h further highlights ongoing retinal inflammation post-blast. The early *IL-10* increase suggests an initial counter-inflammatory mechanism, while its reduction at 24 h may indicate a limitation in the sustained anti-inflammatory response post-blast. The temporal response of *COX1* and *COX2* in the retina post-blast suggests a complex regulatory role in modulating inflammation over time, potentially reflecting attempts to mitigate prolonged inflammation and promote repair. Strategies targeting *COX2* activity during acute phases could mitigate inflammation without completely suppressing the protective aspects of *COX1* activity during later stages.

Our results correlate with those of Wagner et al.,^34^ who observed increased *TLR2* mRNA expression beginning 2 h after a retinal ischemia/reperfusion injury and persisting for up to 7 days, with *TLR3* upregulation occurring at later time points. This suggests that *TLR2* plays a role in mediating the early immune response to retinal damage. Their study also noted an early release of pro-inflammatory cytokines following retinal ischemia/reperfusion injury. Hangai et al.^35^ identified retinal ganglion cells (RGCs), endothelial cells, and neutrophils as sources of *IL-1β*, highlighting their potential involvement in ocular injuries. Mohan et al.^24^ observed RGC damage after bTBI in a mouse model. These findings align with our observations of early upregulation of *COX* genes and cytokines, which likely drive the inflammatory response, highlighting the complex and context-dependent roles of various *TLR*s in retinal injury and the subsequent inflammatory process.

Understanding these temporal expression patterns provides valuable insights into the molecular mechanisms of bTOI and highlights potential therapeutic benefits. The early upregulation of markers suggests that modulating multiple *TLR*s and *COX* enzyme activities could mitigate acute inflammation and ameliorate retinal damage after blast exposure. Therapeutic agents capable of inhibiting multiple *TLR*s may offer more effective protection than those targeting a single *TLR*, as through multiple sites of action, multi-*TLR* inhibitors can modulate multiple early pro-inflammatory signals and reduce downstream damage. Additionally, the significant role of *COX2* in retinal inflammation indicates that *COX2* inhibitors could serve as valuable adjuncts for reducing oxidative stress and promoting tissue repair. The efficacy of these interventions would depend on their timing and alignment with the observed temporal expression patterns, emphasizing the importance of precisely timed therapeutic strategies.

In conclusion, our results suggest that multiple *TLR*s, *COX*s, and *IL*s are upregulated in the retina post-blast and that the use of therapeutic agents that can inhibit multiple inflammatory pathways may be more broadly efficacious than using a single inhibitor to treat blast-induced ocular dysfunction. Our study highlights the dynamic and complex molecular responses in the retina following blast exposure, providing a foundation for developing targeted therapies to alleviate retinal damage and improve outcomes for individuals affected by bTOI. Understanding and modulating the intricate balance between pro- and anti-inflammatory signals are vital for improving these outcomes. While our study provides valuable insights, it has limitations. The use of only male ferrets limits generalizability, and the focus on gene expression provides a snapshot but not the complete picture of molecular changes. Further research is needed to elucidate detailed signaling pathways, long-term functional outcomes, and protein-level and histological changes. Investigating therapeutic interventions targeting these pathways in pre-clinical models is essential for translation.

## Disclaimer

Material has been reviewed by the Walter Reed Army Institute of Research. There is no objection to its presentation and/or publication. The opinions or assertions contained herein are the private views of the author and are not to be construed as official, or as reflecting true views of the Department of the Army or the Department of Defense. The research was conducted under an IACUC-approved animal use protocol in an AAALAC International-accredited facility with a Public Health Services Animal Welfare Assurance and in compliance with the Animal Welfare Act and other federal statutes and regulations relating to laboratory animals.

## Abbreviations Used

ABS: advanced blast simulator
bTOI: blast-induced traumatic ocular injury
*COX*: cyclooxygenase
*IL*: interleukin
qRT-PCR: quantitative real-time polymerase chain reaction
RGC: retinal ganglion cell
SEM: standard error of mean
TBI: traumatic brain injury
*TLR*: Toll-like receptor
